# COEXISTENCE OF PAPULONECROTIC TUBERCULIDE WITH LICHEN SCROFULOSORUM

**DOI:** 10.4103/0019-5154.60367

**Published:** 2010

**Authors:** Jayanta Kr Das, Sujata Sengupta, Subhabrata Mitra, Asok Kr Gangopadhyay

**Affiliations:** *Department of Dermatology, VIMS, Ramakrishna Mission Seva Pratisthan, 99, Sarat Bose Road, Kolkata - 700 026, India.*; 1 *Department of Dermatology, B P Poddar Hospital and Medical Research Centre, New Alipore Block-G, Kolkata - 700 053, India.*; 2 *Department of Dermatology, 15, Girish Chakraborty Lane, Khagra, Murshidabad, West Bengal, India.*

**Keywords:** *Lichen scrofulosorum*, *papulonecrotic*, *tuberculide*

## Abstract

Tuberculides, the supposedly immunologic reactions to the products of dead *Mycobacterium tuberculosis* bacilli deposited in the skin from distant foci of tubercular infection, are presently considered to be of two types-papulonecrotic tuberculide and lichen scrofulosorum. Simultaneous occurrence of both the types in the same patient is very rare. We report the case of an adult male without any known internal tubercular focus who showed two types of skin lesions, clinically typical and histopathologically consistent with the diagnoses of papulonecrotic tuberculide and lichen scrofulosorum, occurring simultaneously. Polymerase chain reaction showed the presence of *Mycobacterium tuberculosis* DNA in papulonecrotic tuberculide type of lesion, and both types of lesions responded promptly to anti-tubercular drugs.

## Introduction

Tuberculides are skin lesions appearing due to hypersensitive reaction to mycobacterial antigen that may indicate tuberculous infection, often occult, elsewhere in the body.[1] The most common sites of infection are lymph nodes. Stain for acid-fast bacilli (AFB) and culture for mycobacteria from the skin lesions are negative.[1] Mantoux test for tuberculosis is positive, and the lesions heal on antituberculous therapy. Using polymerase chain reaction (PCR) technique, mycobacterial DNA has been identified in some lesions.[2] It is conceived that tuberculides are immunological reactions to degenerate dead bacilli or antigenic fractions thereof, deposited in the skin and subcutis.[1]

## Case Report

**Figure 1:**
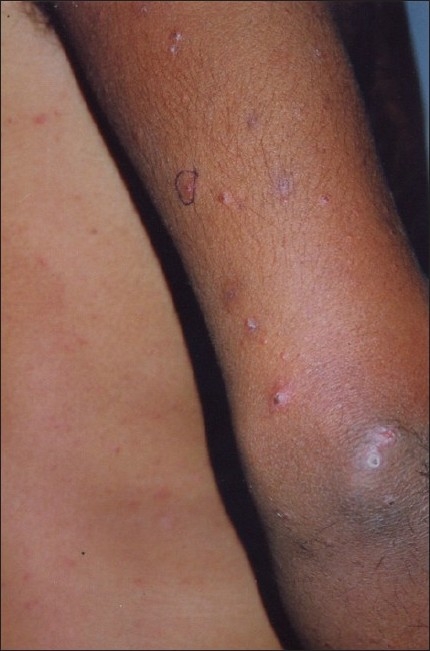
Lesions of papulonecrotic tuberculide on elbows and back

**Figure 2:**
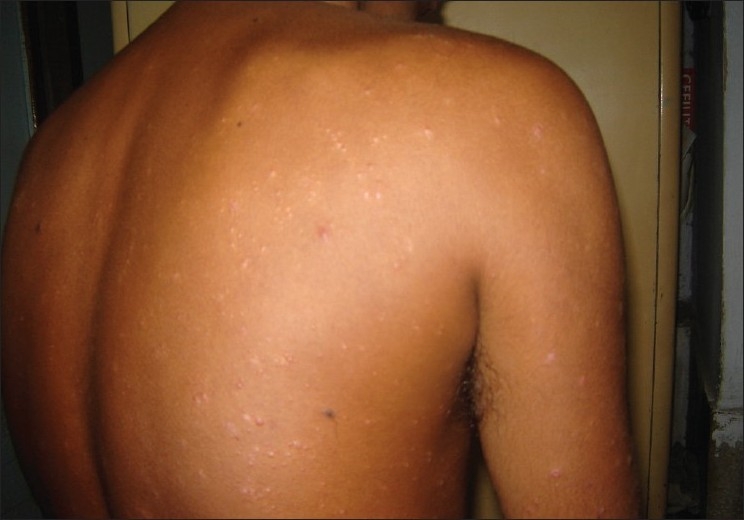
Lesions of papulonecrotic tuberculide and grouped horny papules of lichen scrofulosorum on back

**Figure 3:**
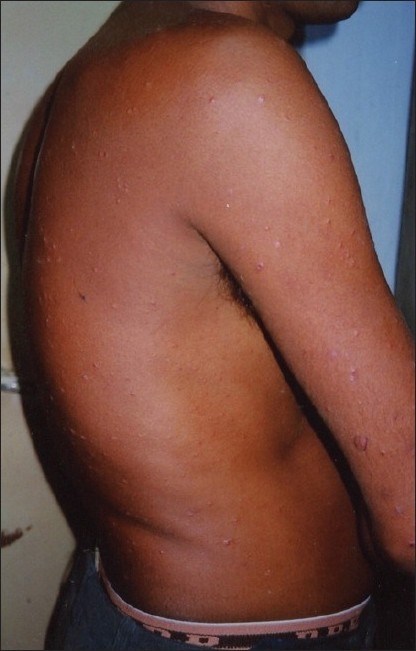
Lesions of papulonecrotic tuberculide and grouped horny papules of lichen scrofulosorum on back and lateral aspect of the trunk

**Figure 4:**
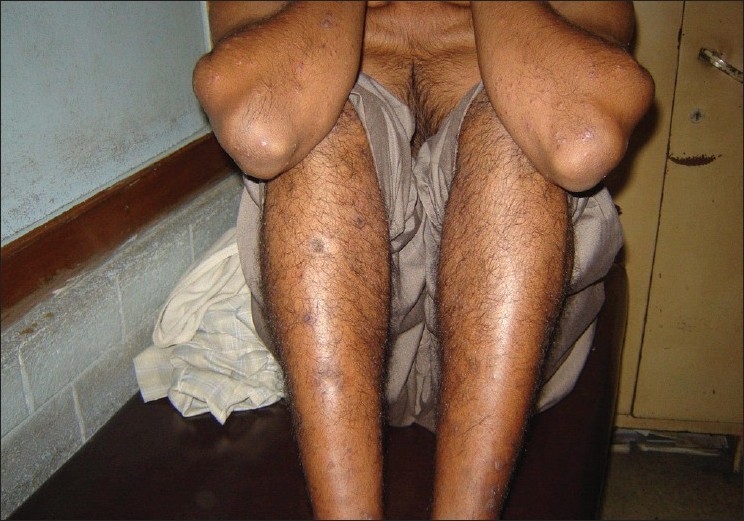
Lesions of papulonecrotic tuberculide on the legs and elbows

**Figure 5:**
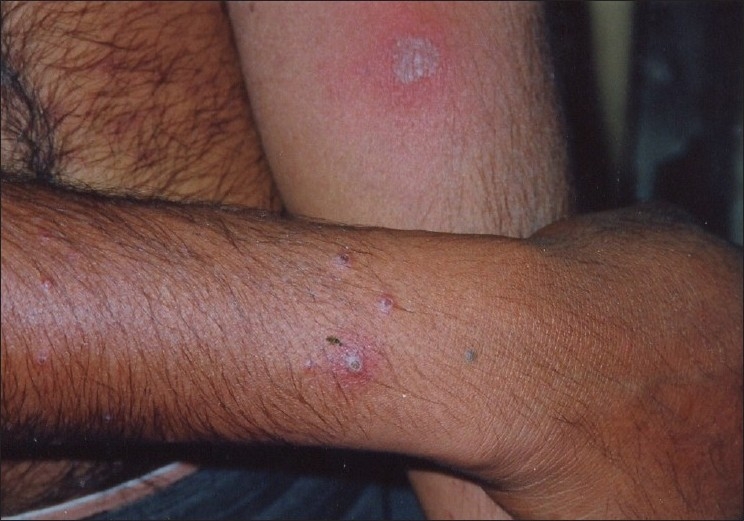
Lesions of papulonecrotic tuberculide and positive mantoux test

**Figure 6:**
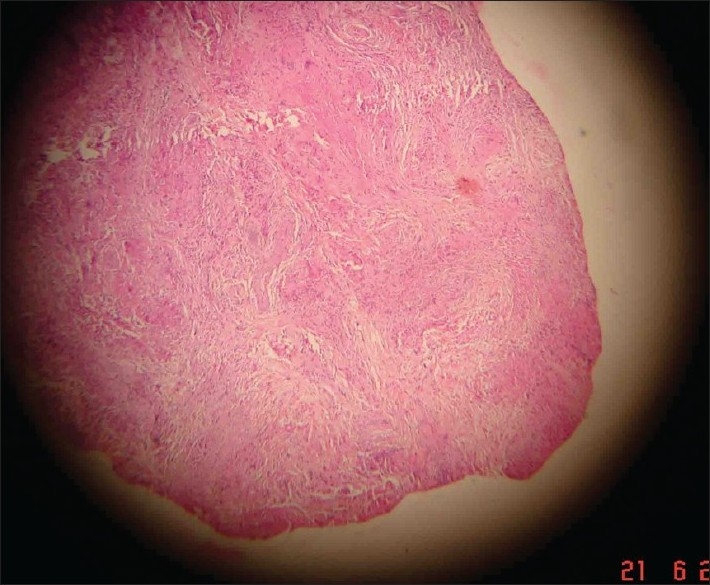
Histopathology of a papulonecrotic tuberculide lesion on the back, showing perivascular mononeuclear infiltrate with multiple granuloma in the mid-dermis, and loss of epidermis in the center (H and E, ×10)

**Figure 7:**
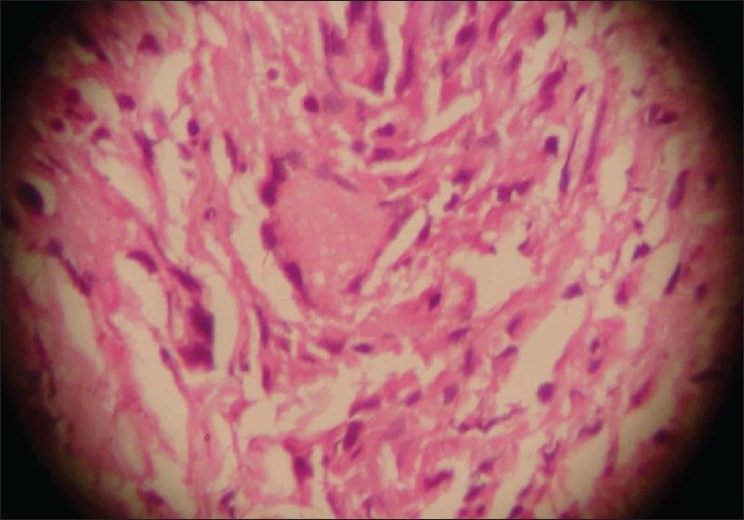
Histopathology of a papulonecrotic tuberculide lesion on the back, showing mononeuclear infiltrate with giant cells (H and E, ×40)

**Figure 8:**
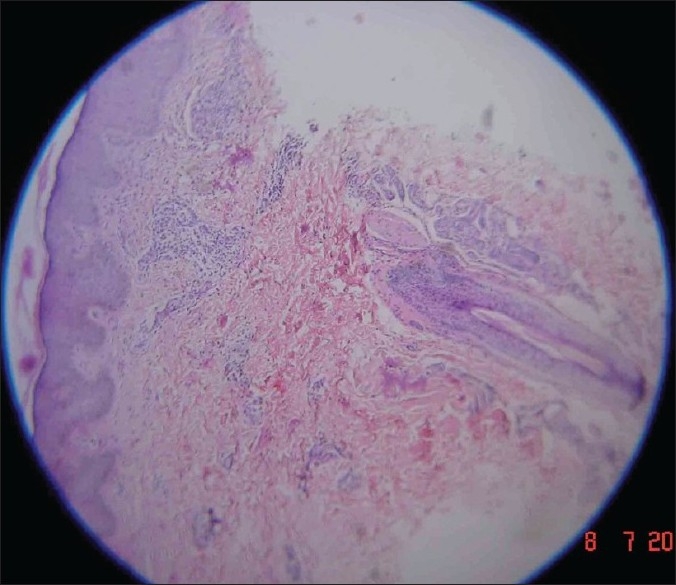
Histopathology of a lichen scrofulosorum lesion on the back, showing granulomas formation in upper dermis and a hair follicle with perifollicular infiltrate (H and E, ×10)

A 29-year-old male presented to us with extensive and symmetric eruption of small papules involving the back, extremities, buttocks, and to a lesser extent, the chest and abdomen. There was definite predilection for extensor surface [Figures 1–4]. The lesions came as recurrent crops for the past three months. There was mild itching but no pain. On examination, the papules were erythematous with size varying from 2 mm to 1 cm in diameter. At the center of some of the papules, there was necrosis of the epidermis and an adherent scale, giving them an umbilicated appearance. In the vicinity of the papules there were a few small pitted scars that apparently evolved at the site of the older papules that healed earlier. On the back, in addition to the papules and scars, there were rather inconspicuous grouped pinkish follicular papules with horny spines on their top [Figures 2 and 3]. The horny papules were distributed in five to six groups, each group forming a rough discoid plaque. Rest of the cutaneous examination was normal. There was no lymphadenopathy. General examination was non-contributory. Mantoux test was strongly positive, with induration of 16 mm, and ulceration [Figure 5]. Barring moderately raised ESR (27 mm), hemogram was normal. Chest x-ray showed no significant abnormality. Histopathology of the skin biopsy of a papule with central necrosis showed evidence of lymphocytic vasculitis. It also showed collections of lymphoid cells and epithelioid cells with rare giant cells of the Langhans type in the upper dermis [Figures 6 and 7]. AFB (Ziehl-Neelsen) stain failed to demonstrate any AFB. This histopathological picture was consistent with papulonecrotic tuberculide (PNT). Histopathology of the grouped follicular horny papules on the back showed hair follicle infiltrated with mononuclear cells and occasional epithelioid cells, and upper dermal granuloma formed by mononuclear cells and epithelioid cells [Figure 8]. Ziehl-Neelsen stain failed to demonstrate any AFB. These lesions were diagnosed as lichen scrofulosorum (LS). PCR technique showed *Mycobacterium tuberculosis* DNA in frozen sections of papulonecrotic tuberculide lesions, but no *M. tuberculosis* DNA was found in lichen scrofulosorum lesions. The patient was put on the standard DOTS (Directly Observed Treatment-Short course) antituberculous treatment (category III) as recommended by the World Health Organization. The patient was administered rifampicin 450 mg plus isoniazide 600 mg plus pyrazinamide 1500 mg three days a week for the first two months, followed by rifampicin 450 mg plus isoniazide 600 mg three days a week for next four months.

The lesions began to heal in two weeks, and by the end of three months there was not a single papule of PNT or LS. There were, however, some pitted scars at the sites of PNT lesions. At the end of six months, the patient was assumed to be adequately treated, DOTS was stopped and the patient was discharged from treatment.

## Discussion

The term tuberculide was first proposed in 1896 by Darier.[1] Historically it contained many entities such as lupus miliaris disseminatus faciei, which has later been firmly categorized as of non-tubercular origin, and other entities like erythema induratum (EI), with which the link of tuberculosis is at best tenuous.[3] According to many authors, the term tuberculide should be meant to comprise of two entities, namely papulonecrotic tuberculide and lichen scrofulosorum.[4,5] According to others, tuberculides comprise PNT, LS, EI, and some other less distinct entities as well.[6,7] The exact steps involved in the pathogenesis of tuberculides remain to be elucidated, but the concept that tuberculides are immunological reactions to degenerate dead bacilli or antigenic fractions thereof, deposited in the skin and subcutis, is largely accepted.[1] That similar skin lesions may occur due to antigenic assault by other mycobacteria is demonstrated by the reported cases of both PNT and LS occurring as reactions, in different patients, to BCG vaccination.[8]

The reaction pattern in a human body to a specific antigenic stimulus is highly individualized, and the reason why a patient with tuberculosis somewhere in the body develops PNT while another develops LS, and why most of the others do not develop either, is not known. Each of these two clinical patterns is not totally homogenous either; for example PNT lesions may be of different sizes, and with different degrees of necrosis or granuloma formation. However, in one individual almost always one kind of tuberculide lesion occurs all the time. So far there have been very few reports of cases with both the types of tuberculides presenting in the same patient at the same point of time.[9] In 1998, Park YM *et al*. reported a case with concomitant LS and EI, and their literature survey yielded 13 cases reported with concomitant PNT and EI, and two cases of PNT associated with erythema nodosum.[7] There is also report of PNT transforming into LS in a child.[10] But extensive literature search failed to yield a single report, barring that of Wechsler HL *et al*. in 1951, of concomitant PNT and LS in the same patient. Thus this case is unique; for to the best of our knowledge, in the last fifty years there has not been a single report of similar case. Histologically, the pictures were consistent with their clinical diagnoses, although not very typical of them. Presence of *Mycobacterium tuberculosis* DNA in PCR in papulonecrotic tuberculide type of lesion, and the rapid resolution of both the types with antituberculous therapy, made the diagnosis very certain.
